# DETERMINING FEASIBILITY FOR USE OF GERIATRIC COGNITIVE SCREENINGS BY MEDICAL CASE MANAGERS SERVING OLDER (50+) PLWH

**DOI:** 10.1093/geroni/igac059.1044

**Published:** 2022-12-20

**Authors:** Monica Maly, Erin Burk-Leaver

**Affiliations:** Colorado School of Public Health, Aurora, Colorado, United States; International Association of Gerontology and Geriatrics, Denver, Colorado, United States

## Abstract

Cognitive impairment (CI) is a significant age-related risk exacerbated by HIV. However, AIDS Service Organizations (ASO) have not implemented effective screening tools for detection of CI in older adults living w/HIV (OALH). This study examined feasibility for use of those tools by medical case managers (MCM) serving OALH. MCMs from nine Colorado ASOs self-administered a questionnaire measuring baseline knowledge of warning signs and risk factors (WS/RF) of CI in OALH, evaluating familiarity with screening of/response to WS/RF of CI within this population. Data from 24 MCMs showed OALH comprised 47% of all clients, and 68% of respondents reported no related training. Similarly, 67% reported not feeling confident identifying WS/RF. 100% of respondents reported not screening for CI, and many endorsed limited awareness/availability of screening tools (87%), referral resources (87%), and training (83%). This identified a need and desire for use/implementation of cognitive screening tools to improve ASO care for OALH.

